# Genomic timetree and historical biogeography of Caribbean island ameiva lizards (*Pholidoscelis*: Teiidae)

**DOI:** 10.1002/ece3.3157

**Published:** 2017-08-01

**Authors:** Derek B. Tucker, Stephen Blair Hedges, Guarino R. Colli, Robert Alexander Pyron, Jack W. Sites

**Affiliations:** ^1^ Biology Department University of West Florida Pensacola FL USA; ^2^ Department of Biology LSB 4102 Brigham Young University Provo UT USA; ^3^ Center for Biodiversity Temple University Philadelphia PA USA; ^4^ Departamento de Zoologia Universidade de Brasília Brasília DF Brazil; ^5^ Department of Biological Sciences The George Washington University Washington DC USA

**Keywords:** anchored phylogenomics, BioGeoBEARs, dispersal extinction cladogenesis, divergence dating, greater antilles, lesser antilles, phylogenetics

## Abstract

The phylogenetic relationships and biogeographic history of Caribbean island ameivas (*Pholidoscelis*) are not well‐known because of incomplete sampling, conflicting datasets, and poor support for many clades. Here, we use phylogenomic and mitochondrial DNA datasets to reconstruct a well‐supported phylogeny and assess historical colonization patterns in the group. We obtained sequence data from 316 nuclear loci and one mitochondrial marker for 16 of 19 extant species of the Caribbean endemic genus *Pholidoscelis*. Phylogenetic analyses were carried out using both concatenation and species tree approaches. To estimate divergence times, we used fossil teiids to calibrate a timetree which was used to elucidate the historical biogeography of these lizards. All phylogenetic analyses recovered four well‐supported species groups (clades) recognized previously and supported novel relationships of those groups, including a (*P. auberi* + *P. lineolatus*) clade (western + central Caribbean), and a (*P. exsul* + *P. plei*) clade (eastern Caribbean). Divergence between *Pholidoscelis* and its sister clade was estimated to have occurred ~25 Ma, with subsequent diversification on Caribbean islands occurring over the last 11 Myr. Of the six models compared in the biogeographic analyses, the scenario which considered the distance among islands and allowed dispersal in all directions best fit the data. These reconstructions suggest that the ancestor of this group colonized either Hispaniola or Puerto Rico from Middle America. We provide a well‐supported phylogeny of *Pholidoscelis* with novel relationships not reported in previous studies that were based on significantly smaller datasets. We propose that *Pholidoscelis* colonized the eastern Greater Antilles from Middle America based on our biogeographic analysis, phylogeny, and divergence time estimates. The closing of the Central American Seaway and subsequent formation of the modern Atlantic meridional overturning circulation may have promoted dispersal in this group.

## 1 INTRODUCTION

Three hypotheses have been proposed to explain the origin of biodiversity across Caribbean islands: vicariance, a temporary land bridge, and overwater dispersal. A vicariance model suggests that the proto‐Antilles were connected to mainland North and South America ~100–70 Ma (Hedges, 2001; Rosen, 1975). The hypothesis of Rosen (1975) has not been supported by geological (Ali, 2012; Iturralde‐Vinent, 2006) or biological (Hedges, 1996b, 2001, 2006; Hedges, Hass, & Maxson, 1992; Williams, 1989) evidence, and the rare cases of ancient Antillean lineages (Noonan et al., 2013; Roca et al., 2004) are of relictual groups and thus problematic (Hedges, 2006). An alternative hypothesis has proposed that a continuous land bridge, GAARlandia, connected the Antilles with the South American mainland 35–33 Ma (Alonso, Crawford, & Bermingham, 2012; Iturralde‐Vinent, 2006; Iturralde‐Vinent & MacPhee, 1999).

GAARlandia coincides with the exposure of the Aves Ridge during an interval of low sea level. There is, however, no firm geologic evidence for a continuous dry connection (Ali, 2012), and any exposed islands of the Aves Ridge would have facilitated overwater dispersal much like the current Lesser Antilles. Comprehensive studies of many groups of organisms, concerning taxonomic composition in the fossil record and living biota (Williams, 1989) and times of origin of lineages (Hedges et al., 1992; Hedges, 1996b; a; Hedges, 2001), have supported overwater dispersal as likely the only mechanism that has operated in the West Indies. For most West Indian amphibians and reptiles studied, this overwater rafting likely initiated from South America (Hedges & Conn, 2012; Heinicke, Duellman, & Hedges, 2007; Reynolds et al., 2013). Although origins of specific groups and way they dispersed to the Caribbean islands have received substantial attention, the colonization patterns among islands in the region remain unclear.

The lizard genus *Pholidoscelis* (Teiidae) includes 21 described species formerly in the genus *Ameiva* (Goicoechea et al., 2016; Tucker et al., 2016). This clade (subfamily Teiinae) is endemic to the Caribbean in the Greater Antilles, Lesser Antilles, and Bahamian Archipelago. Most species are diurnal, active foragers, and feed primarily on insects but occasionally taking bird eggs and small lizards (Schwartz & Henderson, 1991). Because closely related genera are spread across the continental mainland (North, Central, and South America), and species within *Pholidoscelis* inhabit all Greater Antillean and many Lesser Antillean islands, they are an ideal clade with which to test alternative colonization hypotheses of the Caribbean islands.

The phylogenetic relationships and biogeographic history of *Pholidoscelis* are poorly known. An early taxonomic revision of *Ameiva* sensu lato (*Ameiva* + *Pholidoscelis* + *Holcosus* + *Medopheos*) proposed that the Caribbean species formed a single group and likely dispersed from northeastern South America at a hypothesized time when the Antilles were connected to South America (Barbour & Noble, 1915). These authors suggested the observed south‐to‐north gradual transition in morphological characters was evidence against dispersal on flotsam, but this study predated almost all modern ideas about plate tectonics, dispersal, and vicariance biogeography.

**Figure 1:**
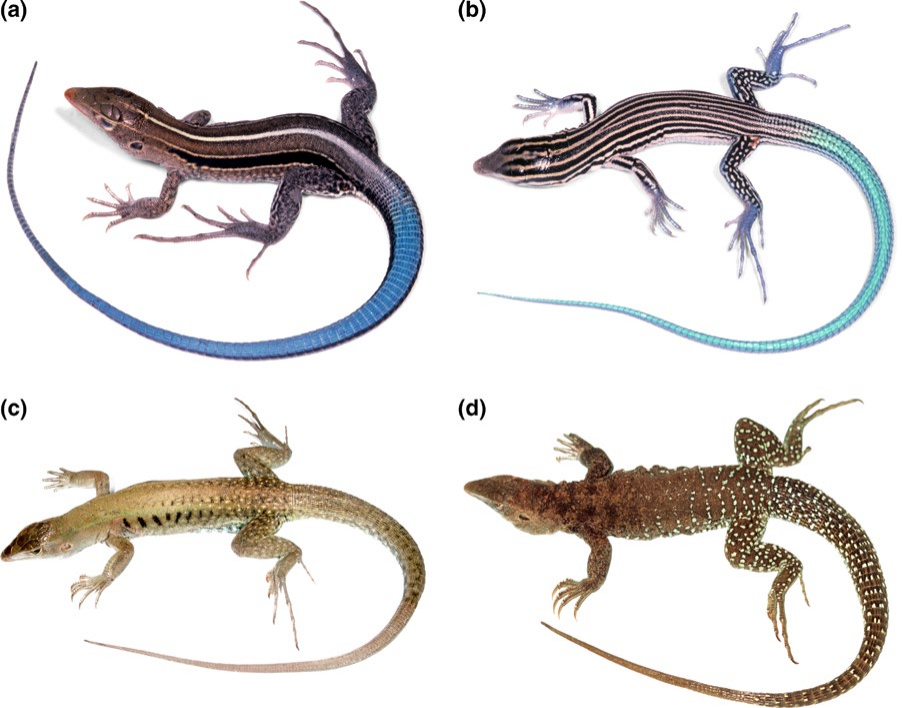
Representative species of the four species groups of Caribbean whiptails of the genus *Pholidoscelis*. (a) *Pholidoscelis auberi of the *Auberi Species Group (U.S. Naval Station at Guananamo Bay, Cuba), (b) *Pholidoscelis lineolatus of the *Lineolatus Species Group (Haiti: l'Artibonite; 1.1 km S Coliming), (c) *Pholidoscelis exsul of the *Exsul Species Group (Puerto Rico: near Arecibo), and (d) *Pholidoscelis plei of the *Plei Species Group (St. Martin: grounds of the St. Maarten Zoo). Photographs by S. Blair Hedges

In the first study to include most species of *Pholidoscelis* since Barbour and Noble (1915), Hower and Hedges (2003) used mitochondrial DNA (12S and 16S ribosomal RNA genes) to investigate the phylogenetic and biogeographic history of the group. These authors recovered a monophyletic West Indian *Pholidoscelis* that included four species groups (Figure 1) and hypothesized a single overwater dispersal event from South America to the Lesser Antilles for this group, followed by speciation in a southeast‐to‐northwest direction. This finding was based on an estimated age of the group at 25–30 Ma, directionality of contemporary ocean currents, and greater species diversity and older clades in the central and eastern islands of the Caribbean.

Hurtado, Santamaria, and Fitzgerald (2014) added the endangered St. Croix ground lizard (*P. polops*) to the existing molecular dataset of Hower and Hedges (2003) to assess its phylogenetic position in the genus and to reevaluate the biogeographic history of the group. These authors argued that the polytomy of the major species groups rejected the previously suggested directional scenario of diversification and hypothesized that both a proto‐Antillean vicariance from the continental mainland (Rosen, 1975), or the GAARlandia land bridge (Iturralde‐Vinent & MacPhee, 1999) were equally plausible to overwater dispersal. Recent systematic studies of teiid lizards have shed further light on the relationships of *Pholidoscelis* (Harvey, Ugueto, & Gutberlet, 2012; Tucker et al., 2016); but an unresolved issue in both molecular and morphological studies has been low nodal support for many relationships, especially those in the backbone of the phylogeny. In this study, we use genomic and mitochondrial DNA datasets to address the phylogenetic and biogeographic history of *Pholidoscelis*. With a combination of molecular and fossil data, we recovered strongly supported relationships within the group and propose alternative hypotheses of the how the genus likely colonized the West Indies.

## 2 MATERIALS AND METHODS

### 2.1 *Pholidoscelis* sampling and laboratory procedures

Of the 21 recognized species of *Pholidoscelis*, we include 15 and 16 for the genomic and mitochondrial datasets, respectively (see Appendix S1 in Supporting Information). Of the five species not included, two of these (*P. alboguttatus* and *P. desechensis*) were until recently considered subspecies of *P. exsul* (Rivero, 1998) and would likely group with this species. Similarly, *P. atratus* was not sampled but at one point was considered a subspecies of *P. pluvianotatus. Pholidoscelis cineraceus* on Guadeloupe and *P. major* on Martinique are both presumed extinct (Schwartz & Henderson, 1991), and tissues are not available for either species. Our phylogenomic dataset of ingroup *Pholidoscelis* included 19 samples representing 15 species with the Central American *Holcosus quadrilineatus* included as the outgroup, based on a previous phylogenomics study (Tucker et al., 2016). Due to increased sampling and existing sequences deposited in GenBank (see Appendix S1), we could augment the in‐group for the mitochondrial dataset to 32 individuals representing 16 species (*P. polops* being the additional species), including many subspecies for some taxa.

We sequenced the mitochondrial gene fragment NADH dehydrogenase subunit 2 (ND2) and estimated gene trees under maximum likelihood (ML) and Bayesian frameworks (see Appendix S2). Phylogenomic data were generated at the Center for Anchored Phylogenetics at Florida State University (www.anchoredphylogeny.com) using the anchored hybrid enrichment methodology described by Lemmon, Emme, and Lemmon (2012). This method uses probes that bind to highly conserved anchor regions of vertebrate genomes with the goal of sequencing less conserved flanking regions. Targeting these variable regions can produce hundreds of unlinked loci from across the genome that are useful at a diversity of phylogenetic timescales. We refer readers to the original paper that generated these data for many species of teiids for additional details (Tucker et al., 2016).

### 2.2 Phylogenetic analyses for *Pholidoscelis*

For the genomic data, we estimated a ML tree with a gamma model of rate heterogeneity from the concatenated dataset of all loci using ExaML v3.0.15 (Kozlov, Aberer, & Stamatakis, 2015) and a parsimony starting tree generated in RaxML v8.1.15 (Stamatakis, 2014). We generated one thousand bootstrap replicate files and Parsimony starting trees in RaxML using a General Time Reversible CAT model of rate heterogeneity (GTRCAT). Replicate files and starting trees were used to produce 1000 bootstrapped trees in ExaML, which were subsequently used to estimate nodal support on our best ExaML tree (see above) using the –z function and GTRCAT model in RaxML.

In addition to the concatenated analysis, we also estimated species trees in MP‐EST v1.5 (Liu, Yu, & Edwards, 2010) and ASTRAL‐II v4.7.9 (Mirarab & Warnow, 2015) due to the possible discordance between any gene tree and its species tree. For the MP‐EST analysis, 1000 nonparametric bootstrapped gene trees were generated in RaxML v7.7.8 (Stamatakis, 2006) per locus. Topologies were then constructed from the gene trees by maximizing a pseudo‐likelihood function in MP‐EST, and results summarized by constructing a maximum clade credibility tree in the DendroPy package SumTrees (Sukumaran & Holder, 2010), with nodal support being calculated as the frequency at which each node was supported across the gene trees. Nonparametric bootstrapped gene trees generated in RaxML for the MP‐EST analysis were also used to estimate nodal support for the ASTRAL‐II analysis, and the species tree was constructed using the “best” RaxML tree for each locus. This method finds the tree that maximizes the number of induced quartet trees in the set of gene trees that are shared by the species tree.

### 2.3 Divergence time estimation

Due to the lack of fossil *Pholidoscelis* that could be assigned to a node in the phylogeny, we estimated divergence times from the Teiidae dataset of Tucker et al. (2016), which included 316 loci (488,656 bp) for 229 individuals representing 56 species. We are aware of no reliable methods for performing fossil‐calibrated divergence time estimates using hundreds of loci for many terminals, so we reconstructed a chronogram for the Teiidae as follows: We used a partitioned alignment of a subset of the data (i.e., reduced number of loci, one individual per species), and implemented PhyDesign (Lopez‐Giraldez & Townsend, 2010, unpublished data) to estimate phylogenetic signal for individual loci on the topology of the MP‐EST species tree from Tucker et al. (2016). The 40 most informative (i.e., highest phylogenetic signal for the species tree topology) loci were then analyzed in BEAST v1.8 using birth‐death tree priors and uncorrelated lognormal relaxed clocks (Drummond, Suchard, Xie, & Rambaut, 2012). We used the topology from the MP‐EST reconstruction in Tucker et al. (2016) as the starting tree, designated a chain length of 200,000,000 generations, sampled parameters every 20,000 generations for a total of 10,000 trees, and determined the best fit model of evolution for each locus using JModelTest (Posada, 2008). We first used only the most informative loci to facilitate convergence and provide an estimated run time for this large dataset and then ran two different analyses of 40 random loci using identical priors and settings.

Two fossils were used to calibrate nodes: a series of dentary fragments representing an ancestor of living *Tupinambis* (estimated age 21–17.5 Ma; Brizuela & Albino, 2004), and GHUNLPam21745, an ancestor for living cnemidophorines (10–9 Ma; Albino, Montalvo, & Brizuela, 2013). Because “*Tupinambis”’* included the genus *Salvator* at the time of the Brizuela & Albino study, we calibrated the node representing the divergence of the (*Tupinambis* + *Crocodilurus* + *Salvator*) clade from *Dracaena*. Two different prior sets were used to confirm that our analysis was not being significantly influenced by prior selection. We first used a uniform prior with the lower boundary set to 17.5 and the upper boundary set to 86 (based on maximum age of Teiidae, see below) for *Tupinambis*. The estimated age of GHUNLPam21745 was used to calibrate the divergence of (*Kentropyx* + *Cnemidophorus* + *Medopheos* + *Ameiva* + *Holcosus* + *Aspidoscelis* + *Pholidoscelis* + *Ameivula* + *Contomastix* + *Aurivela*) from (*Dicrodon* + *Teius*). We used a uniform prior with the lower boundary and upper boundaries set to 9 and 86, respectively. We then ran a second analysis using exponential priors in place of the uniform priors. For *Tupinambis,* we set the mean to 21.5 and offset to 19.25, and for the cnemidophorines, we used 10 for the mean and 9.5 for the offset. In both analyses, we used a uniform prior for the root of Gymnophthalmoidea (Teiidae + Gymnophthalmidae) at 86–70 Ma based on previous squamate studies (Hedges & Vidal, 2009; Mulcahy et al., 2012; Pyron, 2010). We combined two independent runs in LogCombiner v1.8.0 that had converged on the same space to achieve ESS values above 200. The distribution of trees was analyzed using TreeAnnotator, and node bars represent 95% high prior density limits.

### 2.4 Ancestral area estimation

Historical ranges within *Pholidoscelis* were estimated via a ML approach in the R‐package BioGeoBEARs (Matzke, 2013b). This program infers biogeographic histories from phylogenies via model testing and model choice of how this history may be linked to a phylogeny. We implemented the dispersal extinction cladogenesis model (DEC) (Ree & Smith, 2008) and a DEC+J model to account for area cladograms where the ancestral distributions are maintained in one daughter area but not in the other (Matzke, 2013a, 2014). In an attempt to determine the ancestral area of the most recent common ancestor (MRCA) of *Pholidoscelis*, we used a pruned version of the BEAST chronogram containing in‐group taxa in addition to closely related taxa from Central and South America.

Species were assigned to one or more of the following ten regions: Jamaica (JAM), Cuba (CUB), the Bahamas (BHS), Hispaniola (HSP), Puerto Rico (PRI), southern Lesser Antilles (SLA), central Lesser Antilles (CLA), northern Lesser Antilles (AIB), Middle America (CA), or South America (SA). To reduce model complexity and because the most areas any individual species currently occupies is two, we used this number as our maximum range size in all analyses. We tested six different models and compared model fit using log‐likelihoods (LnL), Akaike information criterion (AIC), and sample‐size corrected AIC (AICc) scores. (1) Relaxed Model: Ancestral area estimations were reconstructed with only the BEAST chronogram and geographic distributions of each species. (2) Relaxed+Dist Model: In addition to input included in the Relaxed Model, distance among geographic areas was included in the estimations. (3) Relaxed+ArAdj Model: Like the Relaxed Model except that regions were required to be within 1000 km of one another for an ancestor to inhabit both. This was accomplished via an areas adjacency file. (4) Relaxed+Dist+ArAdj Model: Both the distance matrix and the areas adjacency files were used in reconstructions. (5) South to North Full Model: A review of Caribbean herpetofauna estimates that 79% of lineages have a South American origin while only 15% dispersed from Middle America and 6% from North America (Hedges, 1996b). Moreover, a South American origin of *Pholidoscelis* has been previously proposed (Baskin & Williams, 1966; Hower & Hedges, 2003; Schwartz, 1970). In consideration of these patterns, we implemented a dispersal multipliers file which allowed dispersal only to the north and west, in line with what would be expected based on the directionality of ocean currents and overwater dispersal from South America. The distance matrix and area adjacency file from models 2–4 were also included. (6) South to North Half Model: Identical inputs as the South to North Full Model except that dispersal was allowed contrary to ocean currents (i.e., south and east) at half the probability of traveling north and west. We measured pairwise distances among regions (in km) using “freemaptools.com” and input these values into a distance matrix, dividing all values by the shortest distance so that the lowest value was 1. This feature of BioGeoBEARs allows dispersal ability to be multiplied by distance to the power *x*.

## 3 RESULTS

### 3.1 Phylogenetic analyses

**Figure 2:**
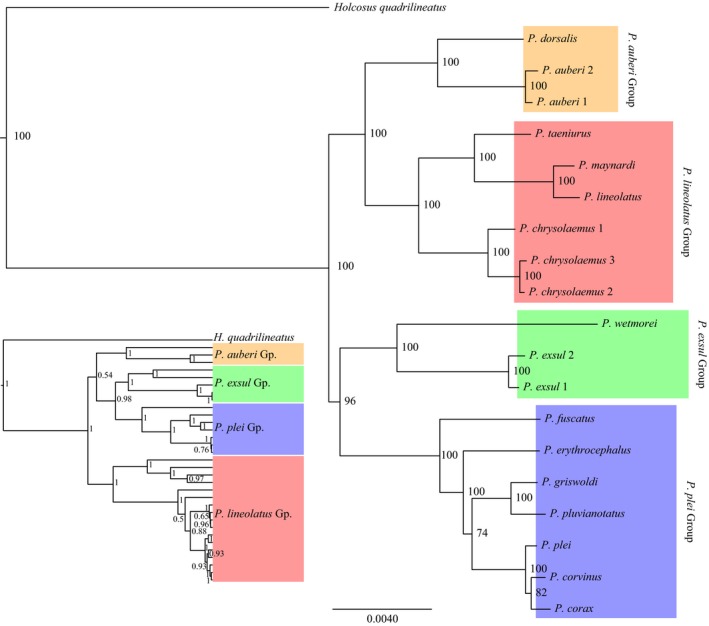
Concatenated maximum‐likelihood analysis of 316 loci (488,656 bp) using RaxML and ExaML. The four species groups of Hower and Hedges (2003) are highlighted with colored boxes for comparison with the ND2 gene tree (see inset; Appendix S3). Values at nodes indicate BS support values and the scale bar represents the mean number of nucleotide substitutions per site

Our ND2 multiple sequence alignment totaled either 1034 bp (protein‐coding only) or 1111 bp (protein‐coding + tRNAs). Sequences are available on GenBank (accession numbers: MF066011– MF066031). The inclusion/exclusion of tRNAs, or the type of analysis (RaxML vs. BEAST), did not have a significant impact on the resulting topologies or nodal support (BEAST analysis including tRNAs shown as inset in Figure 2; for full gene tree see Appendix S3). All analyses recovered the same four species groups proposed by Hower and Hedges (2003); the *auberi* Group (Cuba, Jamaica, Bahamas) containing *P. auberi* and *P. dorsalis*; the *exsul* Group (Puerto Rico region) containing *P. exsul*,* P. polops,* and *P. wetmorei*; the *lineolatus* Group (Hispaniola, Navassa, Bahamas) containing *P. chrysolaemus*,* P. lineolatus*,* P. maynardi*, and *P. taeniurus*; and the *plei* Group (Lesser Antilles) containing *P. corax*,* P. corvinus*,* P. erythrocephalus*,* P. fuscatus*,* P. griswoldi*,* P. plei*, and *P. pluvianotatus*.

While many simulation studies (Mirarab, Bayzid, & Warnow, 2014; Tonini, Moore, Stern, Shcheglovitova, & Ortí, 2015) and empirical datasets (Pyron et al., 2014; Tucker et al., 2016) recover identical or highly congruent tree topologies among concatenated and species tree methods, incongruence has also been noted (Edwards et al., 2016; Streicher & Wiens, 2016). Our nuclear genomic dataset recovered identical topologies with generally high nodal support in both the concatenated (ExaML; Figure 2) and species tree analyses (see Appendix S4; only MP‐EST tree shown because ASTRAL‐II results were identical). However, these relationships differed from those recovered in the mtDNA gene tree. The genomic analyses recovered the deepest divergent event separating the *auberi* and *lineolatus* Groups from the *exsul* and *plei* Groups, whereas the ND2 analysis recovered a (((*P. plei* + *P. exsul*) + *P. auberi*) + *P. lineolatus* Group) topology (Figure 2). The nuclear data recovered the following topologies for the *P. lineolatus* and *P. plei* species groups (the *P. exsul* and *P. auberi* groups only included two species each): *P. lineolatus* Group (*P. chrysolaemus* (*P. taeniurus* (*P. lineolatus* + *P. maynardi*))); *P. plei* Group (*P. fuscatus* (*P. erythrocephalus* ((*P. griswoldi* + *P. pluvianotatus*;* P. plei* (*P. corvinus* + *P. corax*))))).

### 3.2 Divergence time estimation

**Figure 3:**
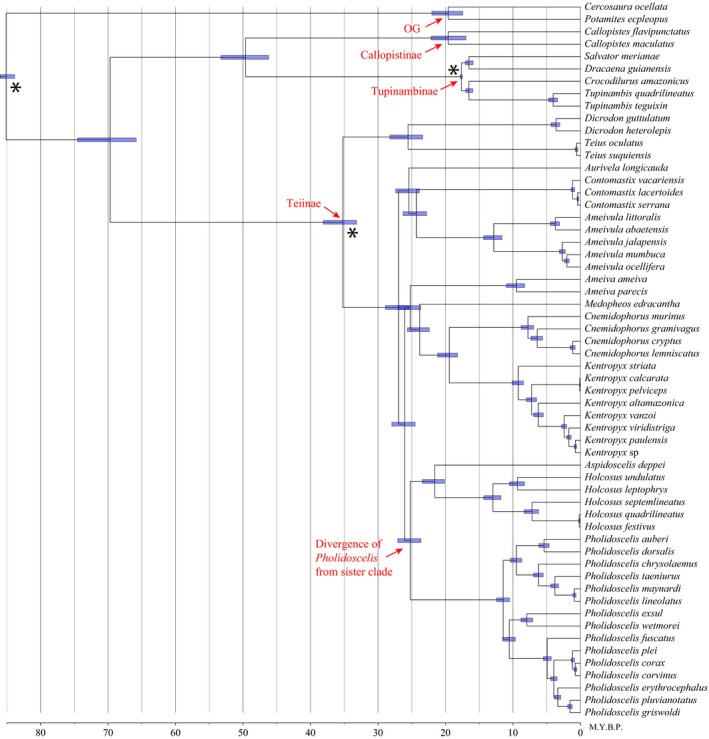
Divergence time estimates of the Teiidae in BEAST using 40 random loci and uniform priors at the calibrated nodes (marked with an *). Scale bar is in millions of years, subfamilies and outgroup taxa are highlighted with red arrows, and node bars are 95% HPD

Here, we present the results of the BEAST analysis using one subset of 40 randomly chosen loci (Figure 3); our other analyses with a different subset of 40 random loci or the 40 most informative recovered identical topologies and similar divergence times. The earliest split in the family occurred 70 Ma and represents the divergence of the small‐bodied Teiinae from all other clades (Tupinambinae + Callopistinae). Our results support a monophyletic Tupinambinae + Callopistinae group, and these two groups began to diverge from one another ~50 Ma. The subfamily Teiinae began diversifying ~35 Ma, with a high concentration of cladogenesis events between 20 and 30 Ma.

The divergence between *Pholidoscelis* and its sister group from Central and North America (*Aspidoscelis* + *Holcosus*) occurred ~25 Ma, with diversification of the former beginning ~11 Ma. The divergence between the *auberi* Group and the *lineolatus* Group occurred ~9.5 Ma, and the divergence between the *exsul* and *plei* Groups occurred ~10.5 Ma. The *Pholidoscelis* topology from the BEAST chronogram is identical to our reconstruction using all 316 loci except for the position of *P. erythrocephalus*. Rather than holding a basal position to a clade containing *P. griswoldi*,* P. pluvianotatus*,* P. plei*,* P. corvinus*, and *P. corax* as in the complete dataset (Figure 2), this species is basal to the (*P. griswoldi* + *P. pluvianotatus*) clade.

### 3.3 Ancestral area reconstructions

**Figure 4:**
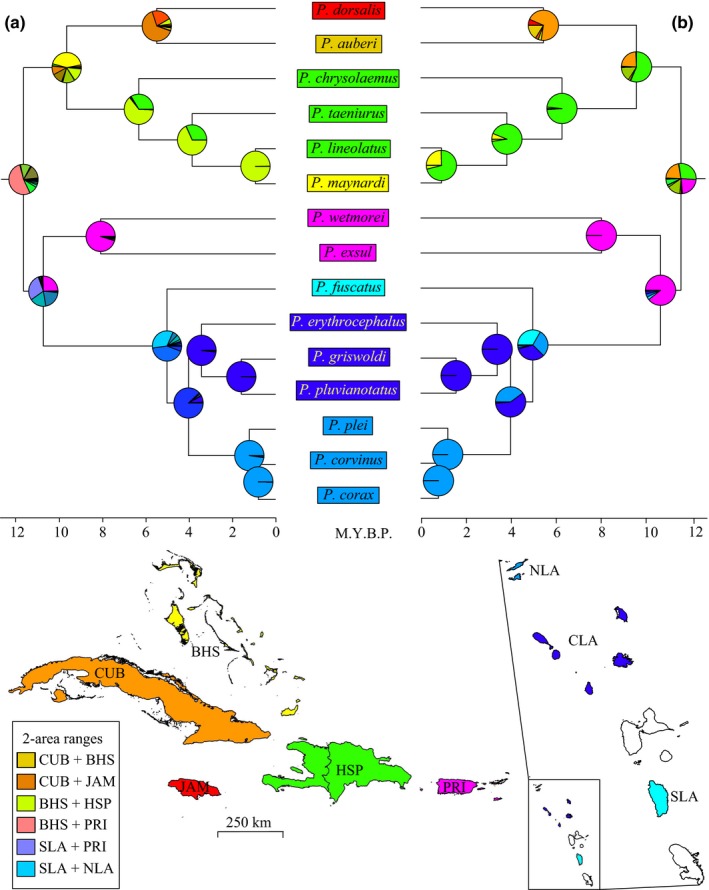
Results of ancestral area estimations in BioGeoBEARs with South American and Central American taxa removed for clarity. a, the dispersal extinction cladogenesis (DEC) reconstruction of the Relaxed+Dist+ArAdj model, where ancestral lineages could disperse in any direction but distance among islands influenced colonization likelihood, and b, the DEC+J reconstruction of the same model. Colors in the pie charts and the boxes highlighting each species match those in the map below to show current distributions and ancestral colonization patterns: Jamaica (JAM), Cuba (CUB), the Bahamas (BHS), Hispaniola (HSP), Puerto Rico (PRI), southern Lesser Antilles (SLA), central Lesser Antilles (CLA), and the northern Lesser Antilles (NLA). Because the max range size was set = 2 in BioGeoBEARs, we also provide additional colors for combinations of 2‐area ranges necessary to interpret the figure

The most likely model under both the DEC and DEC+J was the Relaxed+Dist+ArAdj Model with LnL values of −60.47 and −37.58, respectively (Table 1). In the DEC reconstruction, the ancestor of West Indian *Pholidoscelis* likely colonized the Bahamas or Puerto Rico from Middle America (Figure 4a). The lineage from the Bahamas then colonized the Greater Antilles while the lineage in Puerto Rico colonized the southern Lesser Antilles with subsequent northward dispersal. Under the DEC+J model, however, *Pholidoscelis* likely dispersed from Middle America and began diversification in Hispaniola (Figure 4b). From here, one lineage subsequently colonized Cuba, Jamaica, and the Bahamas, while the other colonized Puerto Rico and the Lesser Antilles.

**Table 1:** Summary of data likelihoods including the log‐likelihoods (LnL), Akaike information criterion (AIC), and sample‐size corrected AIC (AICc) for the six models compared in BioGeoBEARs. Results are shown for reconstructions using the DEC and DEC+J and the best models are designated with an*

	LnL	AIC	AICc
DEC			
Relaxed	−62.31	128.6	129.0
Relaxed+Dist	−62.20	130.4	131.1
Relaxed+ArAdj	−61.89	127.8	128.1
Relaxed+Dist+ArAdj	−60.47*	126.9*	127.6*
South to North Full	−62.09	130.2	130.9
South to North Half	−64.42	134.8	135.6
DEC ± J			
Relaxed	−46.52	99.04	99.77
Relaxed+Dist	−46.13	100.3	101.5
Relaxed+ArAdj	−46.23	98.46	99.19
Relaxed+Dist+ArAdj	−37.58*	83.16*	84.41*
South to North Full	−42.40	92.79	94.04
South to North Half	−39.05	86.09	87.34

## 4 DISCUSSION

Understanding the phylogenetic relationships and biogeographic history of West Indian *Pholidoscelis* has been hampered by poor sampling, conflicting results among datasets, and low nodal support for many clades. We improve upon the most complete studies (Hower & Hedges, 2003; Hurtado et al., 2014) by including *P. corvinus* from Sombrero Island and drastically increasing the amount of molecular data. Using 316 nuclear loci and one mitochondrial gene, we present well‐supported molecular phylogenies of the genus that recover previously named species groups while adding novel insights into the relationships within and among these groups. In addition, with the inclusion of fossil teiids, we provide divergence time estimations for the family and show that divergence between *Pholidoscelis* and the Central American (*Aspidoscelis* + *Holcosus*) clade occurred ~25 Ma, and diversification in the West Indies has occurred over the last ~11.4 Myr. Finally, with an updated phylogeny and chronogram for *Pholidoscelis*, we provide hypotheses on the timing and pattern of colonization of the Caribbean islands. Specifically, our topology and ancestral area estimations show that an ancestor likely dispersed from Middle America and colonized the central part of the Caribbean islands (Hispaniola or Puerto Rico) via overwater dispersal.

### 4.1 *Pholidoscelis* taxonomy

Goicoechea et al. (2016) elevated a subspecies of *Pholidoscelis*,* P. chrysolaemus umbratilis*, to full species based on its clustering with *P. lineolatus* rather than *P. chrysolaemus*. However, the sequence of *P. chrysolaemus umbratilis* (voucher # ALS 156) used by Goicoechea et al. (2016) was published by other authors (Gifford, Powell, Larson, & Gutberlet, 2004) in an earlier study that focused on the subspecies of *P. chrysolaemus*. This earlier study recovered *P. chrysolaemus umbratilis* deeply nested (100% significance level) within *P. chrysolaemus*, and essentially genetically identical to several other subspecies of *P. chrysolaemus*. In addition, the sample of *P. chrysolaemus umbratilis* included here (ALS 143) groups with other *P. chrysolaemus* with a PP of 1 (see Appendix S3). We also performed limited re‐analyses (not shown) using samples in GenBank that suggest that *P. chrysolaemus umbratilis* is indeed a member of *P. chrysolaemus*. Goicoechea et al. (2016) did not explain this discrepancy with previous work, and given our results and the original more comprehensive study of Gifford et al. (2004), we place *P. umbratilis* in the synonymy of *P. chrysolaemus*.

### 4.2 Phylogenetic relationships

The mitochondrial and nuclear datasets (Figure 2, Appendices S3–S4) strongly support the monophyly of the four species groups proposed by Hower and Hedges (2003). The relationships among these groups varied little among phylogenetic methods or the data we used, and here we accept the topology from the phylogenomic dataset (Figure 2), specifically the (*P. auberi* [Cuba, Bahamas, and Jamaica] + *P. lineolatus* (Hispaniola and Bahamas]), and the (*P. exsul* [Puerto Rico region] + *P. plei* [Lesser Antilles]) clades as our working hypotheses. Importantly, the relationships among deep clades revealed here have not been reported previously. Hower and Hedges (2003) proposed a close relationship between the *P. plei* and *P. auberi* groups, and a sister relationship between *P. exsul* and *P. lineolatus* groups. Similarly, Goicoechea et al. (2016) favored a (((*P. plei* + *P. auberi*) *P. exsul*) *P. lineolatus*) topology. Other analyses lack support for monophyletic species groups (Harvey et al., 2012; Pyron, Burbrink, & Wiens, 2013), and a commonality among all of these previous studies has been low nodal support for the backbone of the phylogenies. By drastically increasing the amount of data used in the analyses, we recovered high nodal support for nearly every node in the tree. While increasing the quantity of data has been shown to overestimate nodal support in concatenation analyses, our results are strongly corroborated with species tree methods as well.

### 4.3 Divergence time estimation

We provide a chronogram for the Teiidae estimated with 40 nuclear loci (62,933 bp of aligned DNA), two fossil calibrations, and a third calibration point for the age of the family based on previous studies of squamate reptiles (Figure 3). The only other study to estimate dates for diversification events within the family reported largely similar results to those presented here even though different sources of data and methods were used for the reconstruction (Giugliano, Collevatti, & Colli, 2007), providing evidence that our estimates are appropriate. In comparing results from the two studies, estimated times of deep divergent events differ by 10 Myr or less. Our data estimate ~70 Ma for the age of the node representing the split of the Teiinae subfamily from the remaining clades (deepest split in the family), compared to 63 Ma by Giugliano et al. (2007).

Unfortunately, this earlier study did not include individuals from the West Indies group and sampling in general was limited. Our increased sampling of taxa, loci, and fossils (i.e., GHUNLPam21745), and the application of newer phylogenetic and species tree methods, has improved our understanding of the evolutionary history of the Teiidae.

Hower and Hedges (2003) used a molecular clock approach with protein serum albumin data to estimate divergence times within *Pholidoscelis*. Their estimates are similar to our results; generally speaking, our reconstruction predicts slightly more recent divergence times. For the divergence between *Pholidoscelis* and the Central American *Holcosus*, these authors reported ~26 Ma vs. our 25 Ma, then an age of ~15 Ma for the initial diversification of *Pholidoscelis* compared to our estimate of 11.4 Ma. For the four species groups, Hower and Hedges (2003) provide approximate diversification at 8 Ma (*P. plei* Group), 7 Ma (*P. auberi* Group), 8.5 Ma (*P. exsul* Group), and 11 Ma (*P. lineolatus* Group), slightly older than our estimates for these same events: 4.9 Ma, 5.4 Ma, 7.9 Ma, and 6.2 Ma, respectively. These divergence times clarify the role of overwater oceanic dispersal for *Pholidoscelis* colonization of the West Indies. Our estimate that this group diverged from its sister clade ~25.2 Ma (95% HPD 27.1–23.6; Figure 3) is more recent than dates needed to support other mechanisms (i.e., vicariance, GAARlandia) explaining the biogeographic history of the islands.

### 4.4 Historical biogeography

We compared six models with varying combinations of dispersal probability and distance to better understand how *Pholidoscelis* may have colonized the Caribbean islands. The Relaxed+Dist+ArAdj model best fit the data under both the DEC and DEC+J reconstructions (Table 1). Here, distance among islands was factored into the estimations in addition to disallowing certain combinations of geographic areas based on being separated by more than 1000 km. Although previous research has suggested that nearly 80% of West Indian reptiles and amphibians colonized from South America (Hedges, 1996b), all reconstructions in BioGeoBEARs, except the South to North Full model, suggest a Middle American origin for Pholidoscelis.

This result is not surprising given our topology, in which the sister group to *Pholidoscelis* is the Central America (*Aspidoscelis* + *Holcosus*) clade. Fossil evidence demonstrates that Teiidae inhabited North America and Asia in the Late Cretaceous (Estes, 1983; Sullivan & Estes, 1997), but these early lineages went extinct and living Teiidae have origins in South America (Giugliano et al., 2007; Harvey et al., 2012; Tucker et al., 2016). The almost simultaneous divergences ~25 Ma in our timetree (Figure 3) reveal two hypotheses: (1) the South American ancestor colonized Middle America with subsequent dispersal to the West Indies, or (2) the South American ancestor colonized one of the Caribbean islands with subsequent dispersal to Middle America.

Recent geological studies hypothesize that tectonic collision between South America and Panama may have begun 23–25 Ma (Farris et al., 2011; Montes et al., 2012) much earlier than previous estimates (Webb, 2006). These dates align well with our divergence times separating the Central American and Caribbean taxa from remaining South American Teiidae (Figure 3). This suggests that the Central American Seaway (CAS) separating the continents might have already been fairly narrow, thereby facilitating dispersal of Teiidae into Central and North America. In further support of hypothesis 1 above, the onset of diversification began much earlier in the *Holcosus* + *Aspidoscelis* clade than in West Indian *Pholidoscelis* (Figure 3).

Overwater dispersal from Central America to the Greater Antilles was likely facilitated by air and ocean currents. Modeling studies show that the modern Atlantic meridional overturning circulation (AMOC) was set in motion by the narrowing and eventual closure of the CAS (Maier‐Reimer, Mikolajewicz, & Crowley, 1990; Sepulchre et al., 2014), although it is possible that clockwise currents might have already been in place due to the Coriolis force (Hedges, 2001, 2006). Many have estimated the onset of a proto‐North Atlantic Deep Water at ~12 Ma (Lear, Rosenthal, & Wright, 2003; Poore, Samworth, White, Jones, & McCave, 2006; Sepulchre et al., 2014), which interestingly coincides with the onset of diversification in *Pholidoscelis* (Figure 3). It is possible that a change in ocean currents caused by the closure of the CAS promoted dispersal of these lizards to and among the West Indies.

While the DEC and DEC+J from the Relaxed+Dist+ArAdj model do not correlate exactly on the order in which the islands were colonized, some general patterns are observed. A previous study of *Pholidoscelis* biogeography proposed that the group likely arose by a single overwater dispersal event from South America to the Lesser Antilles, followed by speciation in a southeast‐to‐northwest direction (Hower & Hedges, 2003). This conclusion was based mainly on the directionality of ocean currents and a higher species diversity in the central and eastern portion of the islands compared to Cuba for example. A stepping‐stone model of colonization is not supported in the BioGeoBEARs results nor our phylogenetic tree, rather we see one or more instances of leap‐frog or bypass relationships hypothesized by Baskin and Williams (1966). These authors proposed this scenario based on the observation that *Pholidoscelis* species on neighboring islands were often quite morphologically distinct, like that observed here in the molecular data between the Puerto Rican species (*P. wetmorei* and *P. exsul*) and those from the nearby Anguilla Bank (*P. plei*,* P. corvinus*, and *P. corax*) (Figure 4). Our best‐supported hypotheses suggest ancestral dispersal from Middle America on flotsam and colonization of the Greater Antilles, followed by colonization of the Lesser Antilles (Figure 4). The remaining Greater Antilles and Bahamas were colonized from east to west, as would be expected by ocean and air currents, but the Lesser Antilles were probably colonized from south to north. This would have required a leap‐frog scenario from the eastern Greater Antilles to Dominica, or another island in the southern Lesser Antilles where *Pholidoscelis* is now extinct. Hurricanes affecting the West Indies generally track from east‐to‐west and south‐to‐north, however, occasionally a storm moves in the opposite direction as seen with Hurricane Lenny “Lefty” in 1999 (Hedges, 2006). Further, current systems that flow opposite to the prevailing west‐northwestward Caribbean Current, such as the Cuban Countercurrent (Perez‐Santos, Schneider, & Fernandez Vila, 2015) and the Panama‐Colombia Countercurrent (Andrade, Barton, & Mooers, 2003), could have aided colonization of the Lesser Antilles by *Pholidoscelis*. Although west‐to‐east and north‐to‐south dispersals are relatively rare, they might also be responsible for explaining unusual distribution patterns like those seen in eleutherodactyline frogs (Heinicke et al., 2007) or the reptiles *Anolis longiceps* and *Tropidophis bucculentus* (Hedges, 2001).

Future studies on the geology of the Caribbean region will be extremely valuable in elucidating the biogeographic history of the group. The close proximity of many of these islands to one another suggests that some were connected in the past, but detailed evidence and age estimates for these historic events are lacking, particularly prior to the mid‐Miocene (Iturralde‐Vinent & MacPhee, 1999; Pindell & Kennan, 2009). Due to the relatively recent divergence times in *Pholidoscelis* (i.e., <11 Myr), we propose that most or all colonization events throughout the islands were via dispersal on flotsam and not vicariance. In addition to geological data, the biogeographic history of the group can be improved with the inclusion of extinct species; both those that were recently extirpated: *P. cineraceus* (Guadeloupe) and *P. major* (Martinique), as well as fossil *Pholidoscelis* from La Désirade and Marie‐Galante (both are part of the Guadeloupe island group). Both *P. cineraceus* and *P. major* are represented in museum collections, and methods are now available to isolate sufficient mtDNA for phylogenetic reconstruction from formalin‐preserved animals (Hykin, Bi, & McGuire, 2015). Morphological examination and molecular data from these species can add substantial insight into the history of these lizards.

## AUTHOR CONTRIBUTIONS

All authors conceived the ideas; SBH provided the genetic material; DBT, GRC, JWS, and RAP funded the molecular data collection; DBT and RAP analyzed the data; DBT led the writing; all authors provided feedback and approved the writing.

## CONFLICT OF INTEREST

None declared.
