# Supplementary material for: Genomic timetree and historical biogeography of Caribbean island ameiva lizards (Pholidoscelis: Teiidae)
Source: Ecol Evol. 2017 Aug 1;7(17):7080–90. doi: 10.1002/ece3.3157 (PMC5587475; doi:10.1002/ece3.3157)
Supplement: Supplementary file 1 [file ECE3-7-7080-s001.docx]

*Ecology and Evolution*

**SUPPORTING INFORMATION**

**Genomic timetree and historical biogeography of Caribbean island ameiva lizards (*Pholidoscelis*: Teiidae)**

Derek B. Tucker, S. Blair Hedges, Guarino R. Colli, R. Alexander Pyron, and Jack W. Sites Jr.

**Appendix S1** Voucher and locality data for samples used in this study.

| **Voucher#** | **Genus** | **Species** | **Label in Fig. 1** | **GenBank Accession#** | **Locality** |
| --- | --- | --- | --- | --- | --- |
| **GDC2260** | ***Holcosus*** | ***quadrilineatus*** | ***Holcosus quadrilineatus*** | **MF066031** | **Limon, Costa Rica** |
| **SBH172879** | ***Pholidoscelis*** | ***auberi atrothorax*** | ***P. auberi1*** | **MF066012** | **Cuba: Sancti Spiritus; Trinidad** |
| **SBH161973** | ***Pholidoscelis*** | ***auberi sabulicolor*** | ***P. auberi2*** | **MF066013** | **South Toro Cay, U.S. Naval Station at Guantanamo Bay** |
| **MEG 348** | ***Pholidoscelis*** | ***chrysolaemus*** |  | **EU781099.1** | **Dominican Republic** |
| **SBH194699** | ***Pholidoscelis*** | ***chrysolaemus abbotti*** | ***P. chrysolaemus2*** | **MF066022** | **Dominican Republic: Pedernales Prov.; Isla Beata** |
| **BWMC 06854** | ***Pholidoscelis*** | ***chrysolaemus alacris*** |  | **AY561646.1** | **Dominican Republic, 18°41.36 N, 71°3.692 W** |
| **SBH194588** | ***Pholidoscelis*** | ***chrysolaemus defensor*** | ***P. chrysolaemus1*** | **MF066021** | **Haiti: Dept. du Nord'Ouest; Bombardopolis** |
| **SBH194764** | ***Pholidoscelis*** | ***chrysolaemus fictus*** | ***P. chrysolaemus3*** | **MF066024** | **Dominican Republic: Pedernales Prov.; Cabo Beata** |
| **ALS 83** | ***Pholidoscelis*** | ***chrysolaemus fictus*** |  | **AY561663.1** | **Dominican Republic, 17°49.106 N, 71°25.650 W** |
| **BWMC 6844** | ***Pholidoscelis*** | ***chrysolaemus jacto*** |  | **AY561694.1** | **Dominican Republic, 18°28.251 N, 68°23.997 W** |
| **ALS 188** | ***Pholidoscelis*** | ***chrysolaemus parvoris*** |  | **AY561682.1** | **Dominican Republic, 18°23.10 N, 69°30.00 W** |
| **BWMC 06862** | ***Pholidoscelis*** | ***chrysolaemus regularis*** |  | **AY561649.1** | **Dominican Republic, 19°43.56 N, 71°40.29 W** |
| **ALS 18** | ***Pholidoscelis*** | ***chrysolaemus richardthomasi*** |  | **AY561703.1** | **Dominican Republic, 18°8.10 N, 68°40.00 W** |
| **ALS 143** | ***Pholidoscelis*** | ***chrysolaemus umbratilis*** |  | **AY561676.1** | **Dominican Republic, 18°21.00 N, 71°25.00 W** |
| **SBH266428** | ***Pholidoscelis*** | ***corax*** | ***P. corax*** | **MF066027** | **Anguilla: Little Scrub Island** |
| **SBH269165** | ***Pholidoscelis*** | ***corvinus*** | ***P. corvinus*** | **MF066028** | **Sombrero Id** |
| **SBH194921** | ***Pholidoscelis*** | ***dorsalis*** | ***P. dorsalis*** | **MF066025** | **Jamaica: Kingston** |
| **SBH172686** | ***Pholidoscelis*** | ***erythrocephalus*** | ***P. erythrocephalus*** | **MF066014** | **St. Kitts: Godwin Gut** |
| **SBH190726** | ***Pholidoscelis*** | ***exsul*** | ***P. exsul1*** | **MF066015** | **Puerto Rico: Guanica** |
| **BYU50306** | ***Pholidoscelis*** | ***exsul*** | ***P. exsul2*** | **MF066029** | **18° 25.195'N 64° 37.137'W (Tortola Island)** |
| **BYU50362** | ***Pholidoscelis*** | ***exsul*** |  | **MF066030** | **18° 25.195'N 64° 37.137'W (Tortola Island)** |
| **SBH194215** | ***Pholidoscelis*** | ***fuscatus*** | ***P. fuscatus*** | **MF066020** | **Dominica; Soufrie`re Estate** |
| **SBH192785** | ***Pholidoscelis*** | ***griswoldi*** | ***P. griswoldi*** | **MF066018** | **Antigua: Great Bird Island** |
| **SBH194700** | ***Pholidoscelis*** | ***lineolatus*** | ***P. lineolatus*** | **MF066023** | **Dominican Republic: Pedernales Prov.; Isla Beata** |
| **BWMC 06855** | ***Pholidoscelis*** | ***lineolatus*** |  | **AY561639.1** | **Dominican Republic, 18°39.019 N, 71°2.038 W** |
| **SBH192970** | ***Pholidoscelis*** | ***maynardi*** | ***P. maynardi*** | **MF066019** | **Bahamas: Inagua; Mathew Town** |
| **SBH266002** | ***Pholidoscelis*** | ***plei*** | ***P. plei*** | **MF066026** | **St. Maarten** |
| **SBH192779** | ***Pholidoscelis*** | ***pluvianotatus*** | ***P. pluvianotatus*** | **MF066017** | **Montserrat: St. Peter; Spring Ghut** |
|  | ***Pholidoscelis*** | ***polops*** |  | **JQ240643.1** | **Protestant Cay, Ruth Island** |
| **SBH104391** | ***Pholidoscelis*** | ***taeniurus*** | ***P. taeniurus*** | **MF066011** | **Haiti: Dept. du Sud-Est; 9.5km E. Jacmel** |
| **SBH190731** | ***Pholidoscelis*** | ***wetmorei*** | ***P. wetmorei*** | **MF066016** | **Puerto Rico: Isla Caja de Muertos** |

**Appendix S2** Sanger sequencing protocols and gene tree estimation of the mitochondrial ND2 gene.

DNA was extracted from liver or skeletal muscle using a Qiagen DNeasy^TM^ Blood and Tissue Kit (Valencia, CA, USA). The mitochondrial gene fragment NADH dehydrogenase subunit 2 (ND2) was amplified via polymerase chain reaction (PCR) using primers L4437 (5’–AAGCTTTCGGGCCCATACC–3’) and H5617b (5’–AAAGTGTCTGAGTTGCATTCAG–3’) with the following reagents: 1.0 μl forward primer (10 μM), 1.0 μl reverse primer (10 μM), 1.0 μl dinucleotide pairs (1.5 μM), 2.0 μl 5x buffer (1.5 μM), 2.0 μl MgCl 10x buffer (1.5 μM), 0.1 μl Taq polymerase (5u/(μ1), and 7.56 μl ultra-pure H_2_O. PCR included an initial denaturation for 2 min at 95˚C, followed by 32 cycles at 95˚C (35 s), 52˚C (35 s), and 72˚C (35 s), with a final extension for 10 s at 72˚C. PCR products were vacuum purified with using MANU 30 PCR plates (Millipore) and resuspended in ultra-pure H_2_O. Purified PCR products were included as template in cycle sequencing reactions that used BigDye Terminator kit v3.1 (Applied Biosystems). Cycle sequencing reactions were purified with Sephadex G-50 Fine (GE Healthcare) and sequenced at the BYU DNA Sequencing Center using an ABI 3730xl DNA Analyzer and edited and aligned with Geneious 6.1.8 (Kearse *et al.*, 2012) and Mesquite 3.04 (Maddison & Maddison, 2015).

Gene trees for ND2 were constructed under both Maximum Likelihood (ML) and Bayesian Inference (BI) frameworks. Because the ND2 region we targeted included both protein-coding and tRNA regions, and there were potential alignment issues with the latter, we performed all analyses with and without the tRNA regions, and the coding region was always partitioned by codon position. We used RaxML v7.5.4 (Stamatakis, 2006) with 200 searches for the best tree under a General Time Reversible + GAMMA + Invariant sites model of evolution (GTR+G+I), and nodal support was calculated using 1000 bootstrap replicates, and BEAST v1.8.0 under a HKY + GAMMA model of substitution (Drummond *et al.*, 2012), for both ML and BI analyses, respectively. We used a strict clock and the speciation: birth-death process for the tree prior, a chain of 200,000,000 generations with parameters logged every 20,000 for a total of 10,000 trees, and posterior probabilities (PP) as a measure of nodal support. The output was analyzed in Tracer v1.6 (Rambaut *et al.*, 2014) to ensure ESS values were above 200, and estimated a maximum clade credibility tree in TreeAnnotator.

**Appendix S3** Bayesian inference analysis of the ND2 gene in BEAST (posterior probability support values at nodes). The four species groups of Hower and Hedges (2003) are highlighted with colored boxes for comparison with Fig. 1.


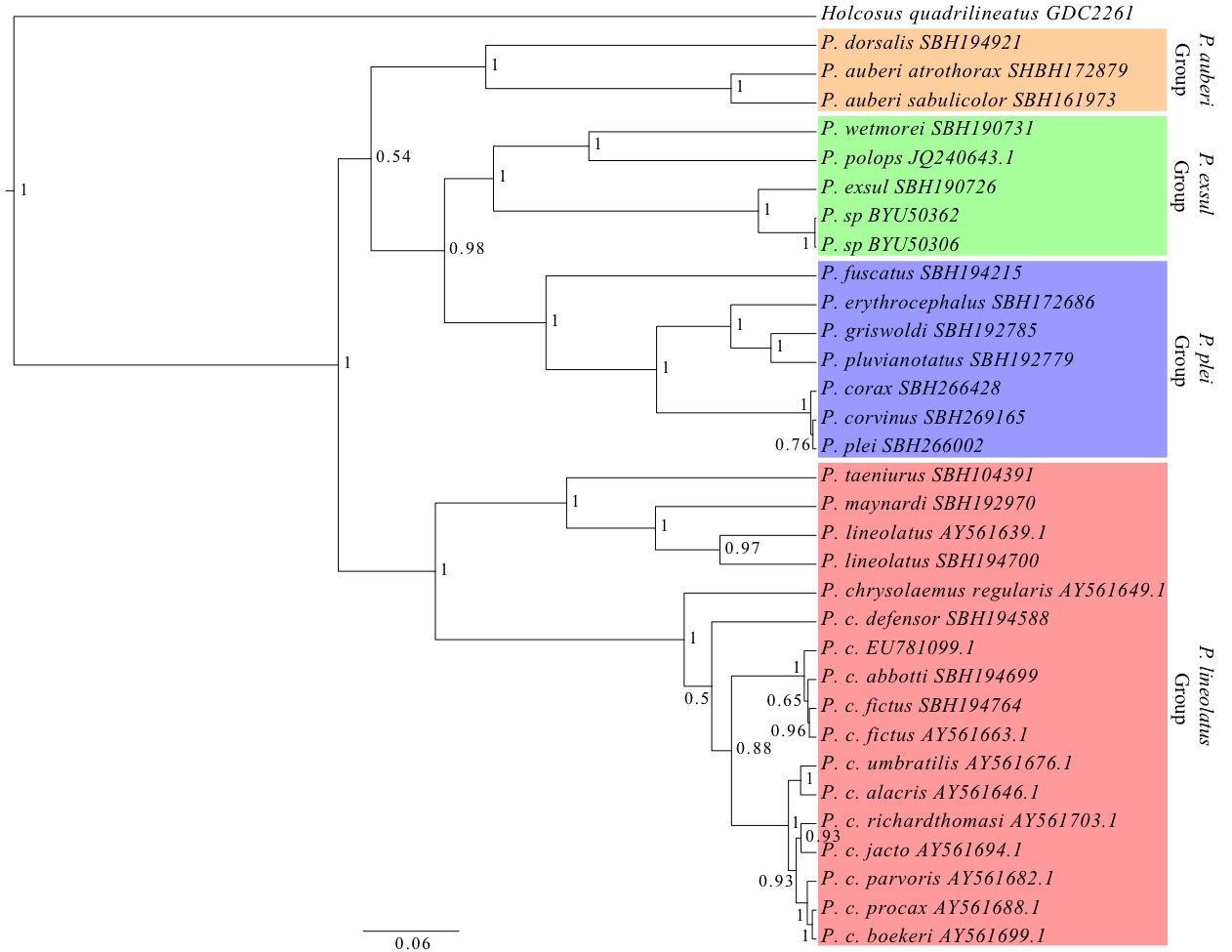


**Appendix S4** Species tree analysis of the genomic data (316 nuclear loci) using MP-EST. Values at nodes indicate the frequency at which that clade was supported across the gene trees. The scale bar represents coalescent units.

References

Drummond, A.J., Suchard, M.A., Xie, D. & Rambaut, A. (2012) Bayesian phylogenetics with BEAUti and the BEAST 1.7. *Molecular Biology and Evolution*, **29**, 1969–1973.

Hower, L.M. & Hedges, S.B. (2003) Molecular phylogeny and biogeography of West Indian Teiid lizards of the genus *Ameiva*. *Caribbean Journal of Science*, **39**, 298–306.

Kearse, M., Moir, R., Wilson, A., Stones-Havas, S., Cheung, M., Sturrock, S., Buxton, S., Cooper, A., Markowitz, S., Duran, C., Thierer, T., Ashton, B., Meintjes, P. & Drummond, A. (2012) Geneious Basic: an integrated and extendable desktop software platform for the organization and analysis of sequence data. *Bioinformatics*, **28**, 1647–1649.

Maddison, W.P. & Maddison, D.R. (2015) Mesquite: a modular system for evolutionary analysis. *Version 3.04* <http://mesquiteproject.org>,

Rambaut, A., A., S.M., Xie, D. & Drummond, A.J. (2014) Tracer v1.6, Available from <http://beast.bio.ed.ac.uk/Tracer>.

Stamatakis, A. (2006) RAxML-VI-HPC: Maximum likelihood-based phylogenetic analyses with thousands of taxa and mixed models. *Bioinformatics*, **22**, 2688–2690.
